# Microarchitecture Influences Microdamage Accumulation in Human Vertebral Trabecular Bone

**DOI:** 10.1359/jbmr.080517

**Published:** 2008-06-02

**Authors:** Monique E Arlot, Brigitte Burt-Pichat, Jean-Paul Roux, Deepak Vashishth, Mary L Bouxsein, Pierre D Delmas

**Affiliations:** 1 Inserm Research Unit 831, Université de LyonLyon, France; 2 Department of Biomedical Engineering, Center of Biotechnology and Interdisciplinary Studies, Rensselaer Polytechnic InstituteTroy, New York, USA; 3 Beth Israel Deaconess Medical CenterBoston, Massachusetts, USA

**Keywords:** microdamage, microcrack, human, vertebral, trabecular bone, microarchitecture, osteoporosis

## Abstract

It has been suggested that accumulation of microdamage with age contributes to skeletal fragility. However, data on the age-related increase in microdamage and the association between microdamage and trabecular microarchitecture in human vertebral cancellous bone are limited. We quantified microdamage in cancellous bone from human lumbar (L_2_) vertebral bodies obtained from 23 donors 54–93 yr of age (8 men and 15 women). Damage was measured using histologic techniques of sequential labeling with chelating agents and was related to 3D microarchitecture, as assessed by high-resolution μCT. There were no significant differences between sexes, although women tended to have a higher microcrack density (Cr.Dn) than men. Cr.Dn increased exponentially with age (*r* = 0.65, *p* < 0.001) and was correlated with bone volume fraction (BV/TV; *r* = −0.55; *p* < 0.01), trabecular number (Tb.N; *r* = −0.56 *p* = 0.008), structure model index (SMI; *r* = 0.59; *p* = 0.005), and trabecular separation (Tb.Sp; *r* = 0.59; *p* < 0.009). All architecture parameters were strongly correlated with each other and with BV/TV. Stepwise regression showed that SMI was the best predictor of microdamage, explaining 35% of the variance in Cr.Dn and 20% of the variance in diffuse damage accumulation. In addition, microcrack length was significantly greater in the highest versus lowest tertiles of SMI. In conclusion, in human vertebral cancellous bone, microdamage increases with age and is associated with low BV/TV and a rod-like trabecular architecture.

## INTRODUCTION

Daily activities subject both cortical and cancellous bone to cyclic loading. The applied loading interacts with the microarchitecture at multiple length scales(1) and forms in vivo microdamage in the form of linear microcracks(2–5) and diffuse damage.(5–8) The propensity of bone to form linear microcracks and diffuse damage varies with age,(9) and the two forms of microdamage have different effects on the mechanical properties of bone.(10,11) Bone remodeling acts to remove microdamage,(12,13) but recent studies showed that the efficacy of damage-initiated remodeling decreases with age.(14) Consequently, with increased age, microdamage may accumulate in load bearing bones and may contribute to the age-related increase in fracture risk.

In particular, human vertebrae sustain compressive loads equivalent to two to three times the body weight during activities of daily living.(15) Furthermore, >1.4 million new vertebral fractures are reported each year,(16) with a large number of these fractures occurring in people with normal BMD.(17,18)

Differences in microarchitecture and microdamage accumulation have been proposed as possible explanations for the overlap in BMD values in patients with and without fracture.(19) Thus, an examination of the interaction between bone microarchitectural parameters and in vivo microdamage in aged human cancellous bone may provide insight into which microarchitectural features are associated with damage accumulation and possibly fracture. To date, only two studies(3,7) have been conducted on human vertebral cancellous bone, and neither of these has examined variables other than age to explain in vivo damage accumulation.

Thus, the primary aims of this study were to (1) describe age-related change in microdamage and (2) determine the relationship between microarchitecture and in vivo microdamage in human vertebral cancellous bone. Because of the high incidence of vertebral fractures,(17) bone from elderly human donors was chosen for this study. Based on reports that microdamage is associated with the loss of mechanical properties of bone(5,19–21) and that bone microarchitecture differs between patients with and without fractures,(22) we hypothesized that microarchitectural features in cancellous bone may influence the age-related accumulation of in vivo microdamage.

## MATERIALS AND METHODS

L_2_ vertebrae from 23 consecutive, recently deceased donors 54–93 yr of age (8 men and 15 women) were used for this study. Specimens were screened using medial-lateral and anterior-posterior high-definition X-rays (Faxitron) to exclude fracture, osteoarthritis, sclerosis, and osteolytic diseases. No additional information regarding donor disease status or medication history was available. Each vertebra was sectioned in half using a Isomet Buehler 4000 microsaw (Buehler) and bulk stained for 11 days at room temperature in 0.5 mM xylenol orange (Sigma-Aldrich) based in 70% ethanol. A cylindrical trabecular specimen ∼8.5 mm in internal diameter was removed in the supero-inferior direction from anterior quadrant in the right half of each vertebrae using a diamond tipped coring tool. The end plate of each core was removed, and the cores were bulk stained for 2 days in 0.5 mM calcein (Sigma-Aldrich) based in 100% ethanol.(23) After DXA and μCT analyses (see below), each core was embedded in methylmethacrylate and cut parallel to the long axis to obtain at least three noncontiguous, parallel, 100 ± 5-μm sections for microdamage evaluation.

### Measurement of trabecular microarchitecture

μCT (μCT40; Scanco Medical, Basserdorf, Switzerland) was used to assess trabecular bone volume and microarchitecture. Images were acquired using a 20-μm isotropic voxel size, subjected to gaussian filtration, and thresholded using an adaptive iterative algorithm.(24–26) Morphometric variables were computed from the binarized images using direct, 3D techniques that do not rely on any prior assumptions about the underlying structure.(27–29) We assessed BV/TV (%), trabecular thickness (Tb.Th, μm), Tb.N (mm^−1^), Tb.Sp (μm), connectivity density (mm^−3^), degree of anisotropy (#), and structure model index (SMI; #), which reflects the rod- versus plate-like nature of the structure. To minimize artifacts caused by pieces of bone remaining from the coring process, the volume of interest excluded the peripheral 1 mm of bone.

### Microdamage analysis

**FIG. 1:**
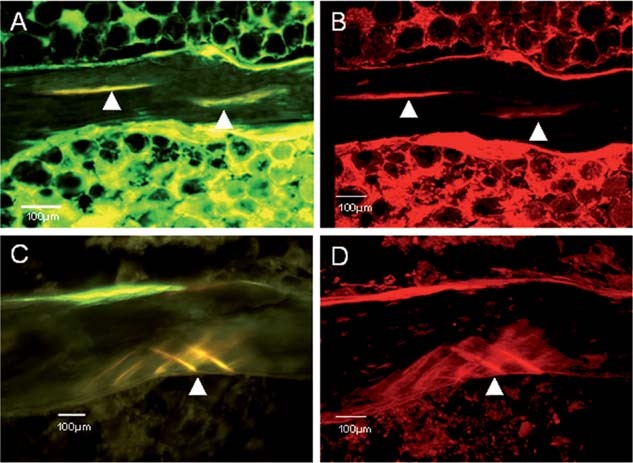
Representative micrographs of a linear microcrack (A and B) and diffuse damage (C and D) under brightfield (A and C) and laser scanning confocal (B and D) microscopy.

Three sections per specimen, containing xylenol orange and calcein-stained microdamage, were measured using fluorescence microscopy at ×200 magnification and morphometry software (Bone Morpho; Explora Nova, La Rochelle, France). Xylenol orange and calcein were observed at excitation/emission wavelength of 440–570/610 and 495/520 nm, respectively. Consistent with ours(23) and other previous studies,(7,11,30,31) microdamage was categorized and quantified as linear microcracks or diffuse damage. Linear microcracks appear as sharply defined lines under the microscope (Fig. 1). In contrast, diffuse damage appears as an area of pooled staining showing submicroscopic cracks under a laser confocal microscope (Leica TCS-SP2; excitation 488 nm/emission 515–566 nm; Fig. 1). Furthermore, the two different morphologies were classified as in vivo or artefactual. Because all vertebrae were bulk stained with xylenol orange and with calcein before and after the section preparation, respectively, the microdamage stained with xylenol orange was considered to be present at the time of donor's death and classified as in vivo. In contrast, microdamage stained with calcein was considered to be produced from drilling and sectioning and was classified as artefactual and not included in subsequent analyses. In vivo microcrack density (Cr.Dn) was significantly greater than artefactual (1.02 ± 0.73 versus 0.41 ± 0.35 #/mm^2^; *p* = 0.003). Moreover, 99.9% of the artefactual microdamage was located in the outer 0.8-mm border of the extracted core; therefore, microdamage in this region was excluded from all analyses.

Outcome assessments included crack density, defined as the number of linear microcracks per bone area (Cr.Dn; #/mm^2^), mean crack length (Cr.le; μm), and diffuse damage density, defined as the number of diffuse damage regions per bone area (Dam.Dn; #/mm^2^) and diffuse damage area, defined as diffuse damage area per bone area (Dam.Ar, mm^2^).

The reproducibility of microdamage identification was assessed by two readers analyzing independently five sections from five different donors. They identified 35 and 32 microcracks, respectively, 31 of which were concordant, providing a κ score of 0.77. The reproducibility of linear microcrack length and diffuse damage area measurements were assessed by two observers on 25 microcracks and/or diffuse damage regions. The intraclass correlation coefficient for both length and area measurements were 0.99.

### Statistical analyses

Because the data for all variables were found to be normally distributed (Kolmogorov-Smirnov tests), parametric tests were used for statistical analyses, except for the comparison between tertiles. Data are presented as the mean and SDn. The following tests were used: paired *t*-test for the comparison between artefactual and in vivo microdamage, Pearson coefficients of correlation for the analysis of the relationships between two variables, regression analysis of the effect of age on microdamage, and stepwise regression for the selection of variables explaining microdamage. The frequency distribution of variables explaining microdamage was also conducted, and the SMI tertiles were used to parse and compare microdamage variables including the length of microcracks and area of diffuse damage (Krusskall and Wallis ANOVA and Wilcoxon *t*-test). For all tests, a level of *p* < 0.05 was required for significance. The statistical analysis was performed using SPSS 12.0 for Windows (SPSS, Chicago, IL, USA).

## RESULTS

### Influence of sex

Overall, there were no significant differences between men and women, except that women tended to be older than men (Table 1). After adjustment for age, women tended to have higher microcrack density (females: 1.145 ± 0.296 #/mm^2^; males: 0.619 ± 0.296 #/mm^2^; *p* = 0.09), lower BV/TV (females: 7.6 ± 2.3%; males: 9.9 ± 3.8%; *p* = 0.10), and lower Tb.N (females: 0.91 ± 0.29 mm^−1^; males: 1.74 ± 0.23 mm^−1^; *p* = 0.09) than men.

**Table 1:** Values of Microdamage and Microarchitectural Variables in Human Vertebral Bone Obtained From Male and Female Cadaveric Donors

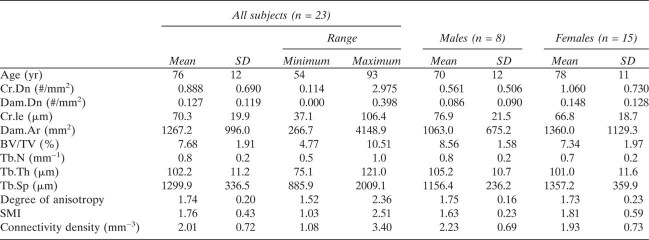

### Influence of age

**FIG. 2:**
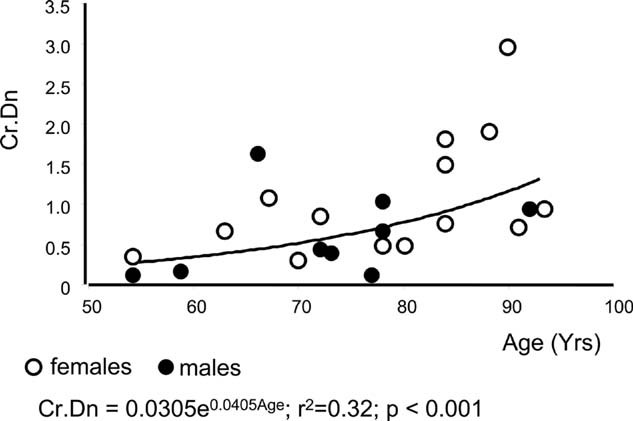
Variation of linear microcrack density as a function of donor age in human vertebral cancellous bone.

**FIG. 3:**
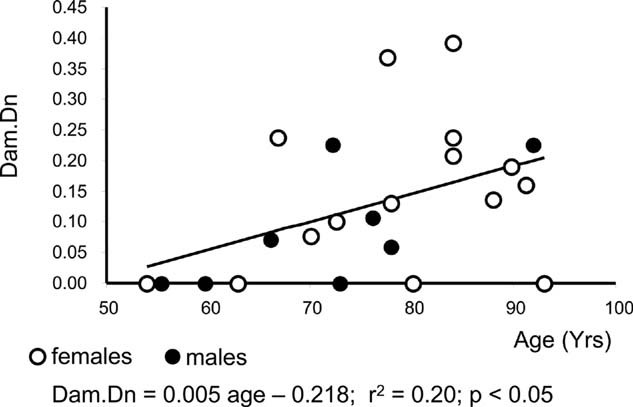
Variation of diffuse damage density as a function of donor age in human vertebral cancellous bone.

Because of the absence of significant sex-related differences in the data set, results from men and women were combined to determine the influence of age. Cr.Dn increased exponentially with age (*r* = 0.55, *p* = 0.01; Fig. 2), whereas Cr.le (*r* = 0.42; *p* < 0.05) and diffuse damage density (*r* = 0.45, *p* < 0.05; Fig. 3) increased linearly with age. BV/TV and Tb.N were negatively correlated, whereas SMI and Tb.Sp were positively correlated with age (Table 2).

**Table 2:** Pearson Correlation Coefficients Between Age, Microdamage, and Microarchitectural Variables Measured From Human Vertebral Cancellous Bone

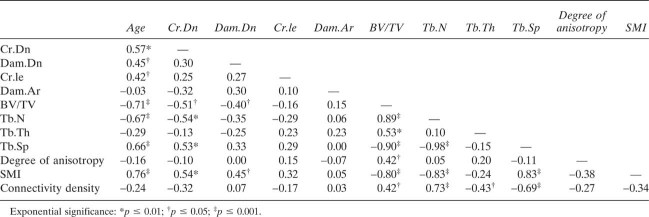

### Relationship between microdamage and trabecular microarchitecture

Microcrack density and diffuse damage density were not correlated to each other but were significantly correlated with bone volume and microarchitecture. Microcrack density was negatively correlated with BV/TV and Tb.N and positively correlated with SMI and Tb.Sp (Table 2). Diffuse damage density was negatively correlated with BV/TV and positively correlated with SMI (Table 2).

**FIG. 4:**
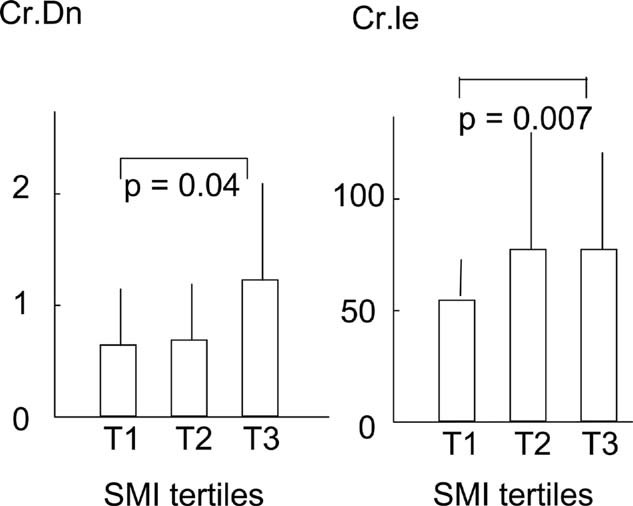
Crack density (left) and crack length (right) according to SMI tertiles in human vertebral cancellous bone.

**FIG. 5:**
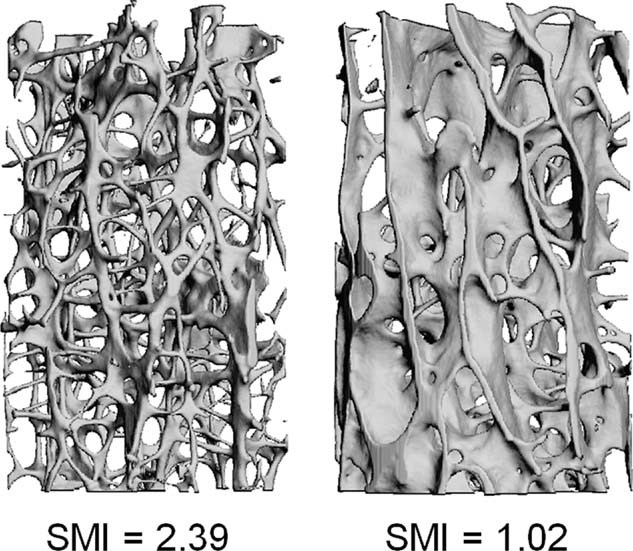
μCT images of two cores of human vertebral cancellous bone, the left one with a high value of SMI (rod-like structure) and the right one with a low value of SMI (plate-like structure).

Stepwise regression analyses indicated that SMI was the best predictor of microdamage accumulation in human vertebral bone. In particular, SMI explained 35% of the variance in Cr.Dn and 20% of the variance in diffuse damage accumulation. Furthermore, when the microdamage data were separated into tertiles based on SMI (first tertile: 1.31 ± 0.20 versus third tertile: 2.27 ± 0.17), microcrack length was significantly higher in the third tertile (no. of donors: 7; Cr.le = 77 ± 52 μm) compared with the first tertile of SMI (no. of donors: 7; Cr.le = 60 ± 31 μm). Similarly, microcrack density was higher (*p* = 0.04) in the third than in the first tertile of SMI. Dam.Ar was higher, but not significantly (*p* = 0.18), in the third (2484 ± 767 μm^2^) than in the first tertile (1013 ± 811 μm^2^). Figure 4 provides a visual representation of microdamage distribution in specimens according to tertile of SMI, and Fig. 5 shows two biopsies with low and high SMI values.

## DISCUSSION

In vivo microdamage and microarchitecture of human vertebral cancellous bone were characterized in this study. We found that linear microcrack density and diffuse damage increase exponentially and linearly with age, respectively. Our results differ from previous reports of age-related microdamage accumulation in human vertebral bone in that previous studies did not find age-related accumulation of linear microcracks or diffuse damage in human vertebral bone.(3,7) Differences in results between our study and previous reports may be attributable to the age distribution of the populations investigated. In the prior studies, both of which used the same samples, the age range of the white subjects was from 25 to 89 yr, but there were only two women ≥70 yr of age. In contrast, our study included a greater proportion of older subjects, with 11/14 women and 6/9 men ≥70 yr of age (Fig. 2). Because much of the increase in microdamage was seen in the oldest specimens, this may explain why age-related changes were not detected in the previous studies. Inclusion of both men and women did not likely affect the outcome of this study because no sex-based differences in the microdamage accumulation in human vertebral cancellous bone were observed here or in previous studies.(3,7)

Age-related accumulation of microdamage found here in human vertebral trabecular bone is consistent with the evidence in human femoral cortical(2) and trabecular bone.(5) Similar to loss in the efficacy of intracortical remodeling process and consequent accumulation of microdamage in cortical bone,(8) the accumulation of microdamage observed here may be explained by a reduction in surface-based remodeling of cancellous bone. A recent study by Waldroff et al.(14) supported this contention because, pursuant to an age-related reduction in bone remodeling, they observed microdamage accumulation in cancellous bone.

Linear microcracks and diffuse damage in bone are associated with different strain modes(1,32) and microstructural features.(1,8,9,33) Furthermore, cortical bone tissue displays a higher threshold of damage formation under compression (4000 μstrain) than in tension (2500 μstrain).(34) Diffuse damage is therefore first to form(9) and, because of a higher degree of material softening,(35) is self-limiting.(36) Thus, there is no a priori basis to suggest that the two forms of damage are correlated. Consistent with the above evidence and with a prior publication,(7) we found no correlation between the two forms of damage in vertebral trabecular bone.

Two recent studies have used in vitro mechanical testing and finite element approaches to investigate the relationship between local microarchitecture and trabecular failure,(20,37) but to our knowledge, this is the first report on the association between in vivo microdamage and trabecular microarchitecture in human vertebral cancellous bone. The degree of inference that can be drawn from microarchitecture is limited in scope because we did not test for the variation on bone material properties in conjunction with structure. For example, damaged trabeculae tend to contain older bone and heterogeneous patterns of mineralization.(33,38)

We found that microdamage, including linear microcrack density, was associated with several aspects of bone microarchitecture, including BV/TV, Tb.Sp, Tb.N, and SMI. Stepwise regression models indicated that SMI was the best predictor of in vivo microdamage. In particular, SMI explained 35% and 20% of the variance in crack density and diffuse damage, respectively. Because SMI is strongly correlated to BV/TV (*r* = −0.80), our finding that, among all the mass and microarchitectural-based variables, SMI is the best predictor of microdamage is somewhat surprising. Our results may have been influenced by our relatively small sample size, which coupled with high variability in microdamage measurements may have limited our ability to identify independent contributors to in vivo microdamage accumulation. Nonetheless, SMI reflects the prevalence of rods and plates in a 3D structure and also provides a measure of the concavity to convexity in a structure.(29,33,39) Thus, for a given amount of bone loss, multiple patterns of bone loss (i.e., different SMI) causing different degrees and morphology of microdamage at the trabecular level can occur. Furthermore, human vertebral trabecular bone with a rod-like structure is in the BV/TV range of <15%, where large deformation bending and buckling(40,41) are predicted to dominate the failure.(42) Hence, changes in SMI, caused by morphological changes at the trabecular level, may play a pivotal role in damage accumulation. For example, the localized resorption of a trabecula can induce stress concentration that may cause initiation and propagation of microdamage.(20) Consistent with the above concept and with the positive correlation between SMI and in vivo microdamage herein, higher SMI has been associated with vertebral wedge fractures in humans(43) and with microdamage formation in older bovine trabeculae.(38) We also found longer microcracks, associated with bone fragility(8,9) in the higher SMI group.

Because accumulation of microdamage in both cortical and cancellous bone tissues has been correlated to loss in bone toughness and strength,(19,44) the accumulation of microdamage reported here may have implications for vertebral fractures. Specifically, the age-related accumulation of microdamage in human vertebral cancellous bone may explain a part of bone fragility associated with vertebral fracture.(45) Further research into factors associated with the formation as well as removal of microdamage in human vertebral bone is therefore warranted.

In conclusion, we found that in vivo microdamage in human vertebral cancellous bone increases exponentially with age and that a rod-like architecture is associated with increased microdamage. Altogether, this observation may contribute to the high incidence of vertebral fracture among the elderly and provides rational for further study of bone remodeling, microdamage, and trabecular microarchitecture at different skeletal sites.
